# Cancer treatments as paradoxical catalysts of tumor awakening in the lung

**DOI:** 10.1007/s10555-024-10196-5

**Published:** 2024-07-04

**Authors:** Emmanuelle Nicolas, Beata Kosmider, Edna Cukierman, Hossein Borghaei, Erica A. Golemis, Lucia Borriello

**Affiliations:** 1 Cancer Signaling and Microenvironment Program, Fox Chase Cancer Center, 333 Cottman Avenue, Philadelphia, PA 19111 USA; 2 Center for Inflammation and Lung Research, Lewis Katz School of Medicine, Temple University, 3500 N Broad St., Philadelphia, PA 19140 USA; 3 Department of Microbiology, Immunology, and Inflammation, Lewis Katz School of Medicine, Temple University, 3500 N Broad St., Philadelphia, PA 19140 USA; 4 Department of Cancer and Cellular Biology, Lewis Katz School of Medicine, Temple University, 3500 N Broad St., Philadelphia, PA 19140 USA

**Keywords:** Dormancy, Extracellular matrix, Neutrophil extracellular trap, Fibrosis, Inflammation, Chemoradiation

## Abstract

Much of the fatality of tumors is linked to the growth of metastases, which can emerge months to years after apparently successful treatment of primary tumors. Metastases arise from disseminated tumor cells (DTCs), which disperse through the body in a dormant state to seed distant sites. While some DTCs lodge in pre-metastatic niches (PMNs) and rapidly develop into metastases, other DTCs settle in distinct microenvironments that maintain them in a dormant state. Subsequent awakening, induced by changes in the microenvironment of the DTC, causes outgrowth of metastases. Hence, there has been extensive investigation of the factors causing survival and subsequent awakening of DTCs, with the goal of disrupting these processes to decrease cancer lethality. We here provide a detailed overview of recent developments in understanding of the factors controlling dormancy and awakening in the lung, a common site of metastasis for many solid tumors. These factors include dynamic interactions between DTCs and diverse epithelial, mesenchymal, and immune cell populations resident in the lung. Paradoxically, among key triggers for metastatic outgrowth, lung tissue remodeling arising from damage induced by the treatment of primary tumors play a significant role. In addition, growing evidence emphasizes roles for inflammation and aging in opposing the factors that maintain dormancy. Finally, we discuss strategies being developed or employed to reduce the risk of metastatic recurrence.

## Introduction

With a total of 2,001,140 new cases and 609,820 cancer deaths estimated to occur in 2024 in the United States alone, cancer remains a major health problem worldwide [1]. While great progress has been made in the treatment of cancer patients, metastatic disease is frequently not amenable to treatment and accounts for the bulk of lethality for most common cancers. With a number of cancer survivors in the United States projected to increase to 22.2 million by 2030, identifying individuals at risk for tumor recurrence, and finding strategies to block that recurrence, is of critical importance.

Metastasis is the endpoint of a complex sequence of events [2, 3]. It starts with a phase of tumor cell dissemination from the primary tumor to either distant sites within the same organ, or to distal organs. The dissemination phase may be followed by a dormancy phase during which disseminated tumor cells (DTCs) do not yet proliferate to form a clinically detectable metastatic lesion. This is because signals coming from the new microenvironment actively restrain the growth of DTCs, while in tandem, changes in the transcriptional programs of DTCs maintains a reversible growth arrest in these cells. These dormant DTCs are clinically undetectable, chemo-resistant, and immune privileged, which allows them to survive for years or even decades [4]. Although there is some overlap between the phenotypes of DTCs and the more recently described drug-tolerant persister cells (DTPs), there are a number of differences (discussed in [5]). Most notably, DTPs are an extremely heterogeneous population of both fast- and slow-proliferating cells that typically are transiently induced by drug treatment and contain metabolic and signaling features that differ from DTCs.

The awakening of dormant DTCs leads to metastatic relapse. The period between primary tumor detection, initial apparent complete response, and metastatic relapse varies between cancer types, and is currently unpredictable in individual patients [6, 7]. Awakening of dormant DTCs and tumor recurrence can be as brief as a few weeks after apparent complete response to treatment, or as long as 20 years or more [8, 9]. For clinicians, dormancy represents a window of opportunity for early intervention [10]. Better understanding of the biology of dormant DTCs, including the nature of the signals that trigger awakening from dormancy, might lead to treatments that prevent metastatic outgrowth by eradicating dormant DTCs and/or by maintaining DTCs in a perpetual dormant state.

Many types of tumor, including breast, colon, head and neck, and renal cancer, as well as neuroblastomas and sarcomas, frequently metastasize to the lung [11], with estimates that as many as 54% of tumors that spread beyond their primary site target this organ [12]. The lung is physically proximal to sites of common tumors such as breast, and the lymphatic and hematogenous systems are enmeshed in lung tissue. DTC access to lung tissue can proceed through blood vessels, lymphatics, or in some cases based on cells directly shed into the pleural cavity. Because of the frequent targeting of the lung by DTCs, the specific mechanisms governing metastatic recurrence in this organ are of high interest. While systemic inflammation plays a major role [13], the mechanisms that mediate metastasis depend, in part, on organ-specific determinants [14, 15], including organ-specific immune responses in which structural cells (e.g., endothelium and fibroblasts) have key roles [16–18].

In this review, we focus on the process of DTC awakening and metastatic outgrowth in the lung, first defining the structural and immune features of the normal lung relevant to metastasis. Emergence from dormancy is typically triggered by changes in the DTC microenvironment and involves innate (such as macrophages, neutrophils and NK cells) and adaptive (T and B lymphocytes) immune cells, and other stromal cells, causing an overt outgrowth to clinically detectable metastases [19]. Importantly, recent literature emphasizes that lungs bearing metastases are often not “normal” for many patients in a real-world setting but have pathological features. Many classic studies of lung function were performed in animal model systems or in healthy young individuals, now appreciated to not effectively simulate conditions in individuals typically suffering from cancer (e.g., [20]). Individuals diagnosed with cancer are typically older; aging is associated with a progressive increase in a low-grade, non-resolving, proinflammatory state (termed inflammaging) [21, 22]. The process of aging causes numerous changes in lung tissue, affecting the abundance and functional properties of essentially all resident cell populations [23, 24]. “Lifestyle”-associated factors can also induce further damage to the lung environment. Although smoking rates are declining, about half of patients with any form of cancer report a history of smoking and 10–18% report current smoking [25–27]. Besides its well-described mutagenic effect, tobacco smoke also alters immune system function, triggering chronic inflammation by a variety of means [28–30]. Many patients who are long-term smokers suffer from chronic lung diseases that grossly alter the cellular composition, inflammatory state, and extracellular matrix (ECM) [27–30]. Rates of obesity are rising globally [31]. Obesity causes chronic low-grade inflammation and leads to changes in the immune landscape of multiple organ systems, including the lung [32–34]. These and other chronic predispositions discussed below simulate low-level wound-healing environment, long known to stimulate cancer cell growth [35]. The recognition of the interaction of these factors will be important help tailor improved treatments for individual patients. Importantly, cancer patients diagnosed with localized or invasive disease, who may already have DTCs in the lung, typically receive chemotherapy, radiation, surgery, and various types of molecularly targeted drugs. As an unintentional side effect, these treatments can impair tumor-specific immunity and promote metastatic outgrowth by inducing fibrosis and inflammation-mediated growth signals [15, 36–40].

## Lung architecture and immunomodulatory signaling

**Fig. 1:**
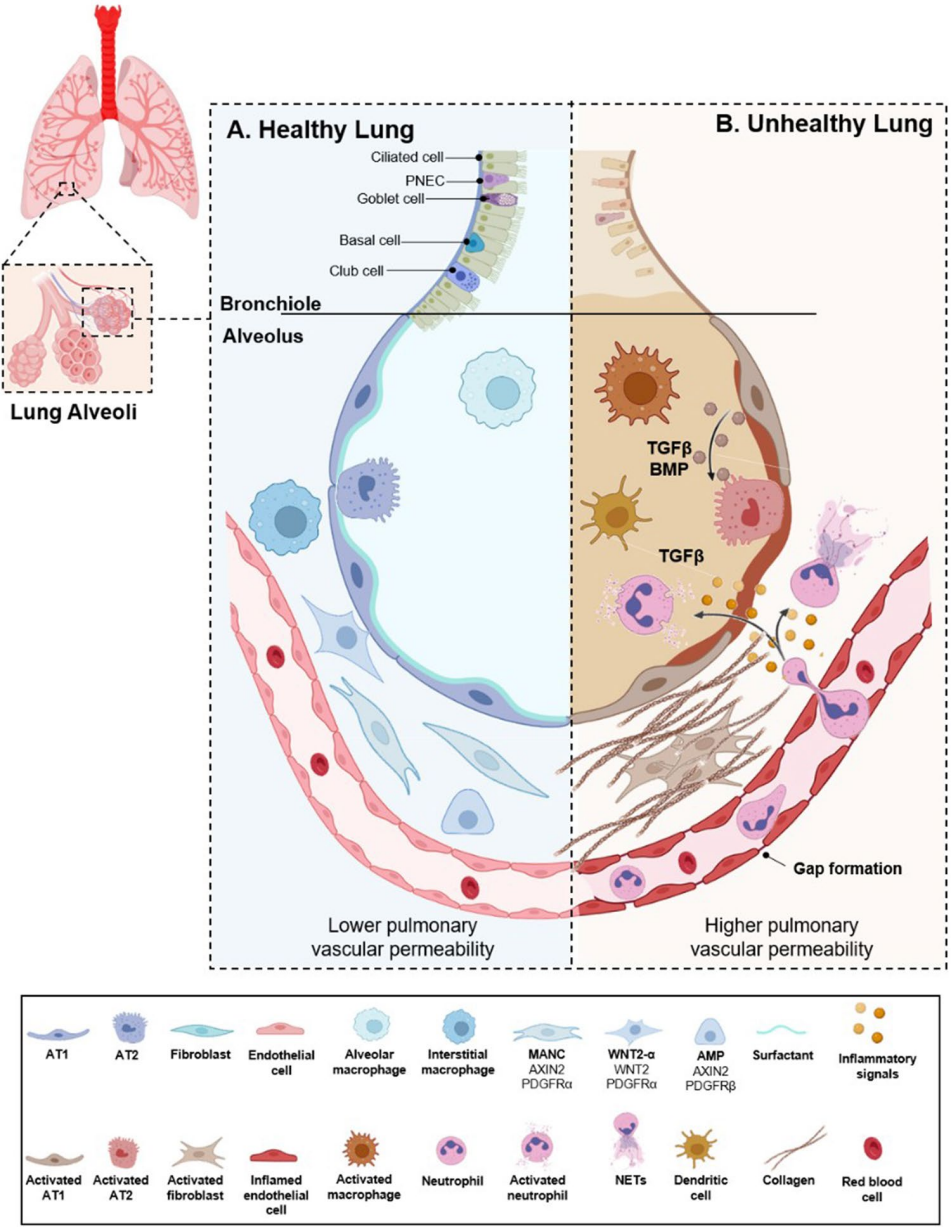
Lung alveoli in health and disease. **A** Air enters and exits the lung through large and small branching airways (the bronchi and bronchioles) that terminate in alveoli, which participate in gas exchange and are surrounded by capillary plexus. The cellular lining of bronchi and bronchioles is complex, including a respiratory epithelium composed of basal cells, multi-ciliated cells, secretory (club) cells, goblet cells, brush (tuft) cells, and others, supported by an underlying layer of stromal cells. The alveoli are lined by two epithelial cell types: alveolar type-1 (AT1) and alveolar type-2 (AT2) cells. AT2s are cuboidal, sparsely distributed among the AT1s, and comprise 5% of the total surface area. AT2s have a unique multi-apical polarity and multi-lumen enfacement organization [47] and secrete surfactant, a lipid-protein complex which decreases alveolar surface tension at the air–liquid interface. AT2s both self-renew and differentiate into AT1s, thereby serving as stem cells. At homeostasis, turn-over is infrequent. However, in settings of lung injury and tissue repair, changes in cell signaling including activation of WNT [48, 49] and reprogramming of the transforming growth factor (TGF) β and bone morphogenic protein (BMP) support AT2 replication and differentiation to AT1 cells. AT1s are very thin, terminally differentiated and have a large surface area covering 95% of the alveolar luminal. AT1s share a basement membrane and mediate gas exchange with the endothelial cells of the pulmonary capillaries. The capillary endothelium is covered with pericytes, which help regulate blood flow [50]. In addition, several types of interstitial fibroblasts comprise a supportive mesenchyme encompassing the alveoli. The pulmonary extracellular matrix (ECM) provides mechanical stability and elastic recoil, which are essential for physiological lung function. Tissue homeostasis is in part maintained by biomechanical functional units made up of resident fibroblasts and self-generated interstitial ECM. These units provide the lung (and other organs) with the needed tensile strength (e.g., collagen) and elasticity (e.g., elastin). Of note, any disturbance or aggravation to the organ is repaired with the participation of these units [35]: through secretion and activation of proteases [51], release or storage of ECM stored factors such as TGFβ that trigger local and systemic changes, as well as the activation of ECM enzymes such as lysyl hydroxylases (LOX), which catalyze crosslinking of collagen and/or elastin fibrils to remodel the fibrous ECM bundles [18, 52–54]. In cases of transient lung injury, ECM proteases and LOX [55] play an important role in the degradation and turnover of all matrix components needed for repair and regeneration, preventing fibrosis [56, 57]. Besides systems required for air exchange, the lymphatic network penetrates the lung, allowing movement of lymphocytes and other immune cells through the tissue, and performing other functions including drainage of interstitial fluid, and removal of cellular debris [58]. The narrow lymphatics drain into larger collecting vessels that are components of bronchovascular bundles and also reside in interlobular septa; these collecting vessels in turn drain to collecting lymph nodes. Immune homeostasis in the healthy lung is maintained through interactions of several cell types, including fibroblast units, resident alveolar macrophages, dendritic cells, alveolar type-1 (AT1), and alveolar type-2 (AT2) cells. Surfactant is composed of surfactant proteins which bind and neutralize viruses. Vascular capillaries line the alveolar walls to facilitate gaseous exchange and infiltration of circulating immune cells. In the interstitial compartment, interstitial fibroblast units, including mesenchymal alveolar niche cells (MANCs), Axin2-positive myogenic precursors (AMPs), and Wnt2-expressing platelet-derived growth factor-α (PDGFRα)-positive cells (WNT2–Pα), comprise a supportive mesenchyme encompassing the alveoli. **B** In pathological conditions, many tissue-resident alveolar macrophages are lost and replaced by bone marrow-derived macrophages. This occurs in parallel with infiltration of inflammatory cells such as neutrophils, recruitment of which is facilitated by chemokines and disrupted barrier integrity. The activated epithelium secretes a plethora of mediators that induce proliferation and activation of fibroblast units, which in turn dynamically modify the interstitial ECM, promoting fibrosis [35]. During lung injury and subsequent tissue repair, activation of WNT signaling and secretion of TGFβ-related factors such as BMP and others support AT2 replication and differentiation to AT1 cells. This figure was created in part using BioRender (BioRender.com)

The primary function of the lung is to perform gas exchange with the circulatory system, removing carbon dioxide and taking up oxygen to support life. In addition, because inhalation is a source of environmental irritants and pathogens, the lung is an important immunological interface. Cell populations resident in or recruited to the lungs employ innate and adaptive strategies to limit and clear infections. In addition, the lung has evolved multiple processes of repair/regeneration/remodeling [41] to preserve homeostasis following tissue damage. The normal functioning of the lung requires complex interactions of many different cell types arranged in a precise architecture and supported by ECM [42–44] (Fig. 1A) although as discussed in detail below, both this architecture and ECM can be significantly disrupted in pathological settings [45, 46], influencing the likelihood of cancer metastasis.

It is important to study DTC dormancy and awakening in the context of lung immune surveillance, as immune cells and secreted cytokines and chemokines play a major role in governing DTC behavior [4]. Immune homeostasis in the healthy lung is maintained through coordination of several pathways. Mucus secreted by goblet cells and submucosal cells of the large and small airways, and surfactant produced by AT2 cells, provide a physical barrier that limits the access of pathogens and other irritants to the lung epithelia, playing an important role in host defenses. Mucus and surfactant have both biophysical and immunomodulatory functions [59]. Besides mucins, which create the characteristic gel-like properties, mucus contains IgA and other immunoglobulins, and antimicrobial proteins, such as lysozyme. Surfactant is composed of a complex mix of dipalmitoylphosphocholine (DPPC) and other phospholipids, cholesterol and other neutral lipids, and surfactant proteins (SP-A, -B, -C, and -D, sometimes called soluble defense collagens), which bind and neutralize viruses and have additional immunomodulatory effects [59, 60].

Application of single cell sequencing and spatial transcriptomics has revealed a strikingly diverse set of tissue resident macrophages, including multiple alveolar and interstitial populations, which is augmented by additional recruited macrophages during inflammation [61]. Acute infection with pathogens or exposure to environmental irritants leads to activation of lung-resident immune cells and the infiltration of immune cells from the periphery (Fig. 1B). Alveolar macrophages are the most abundant immune cells and are the first line of defense [61]. Signaling between alveolar macrophages and epithelial AT2 cells, which communicate with the underlying mesenchymal and endothelial cells, together modulate the activation of the immune system, contributing to both activation and inactivation at the end of an infection [62, 63]. Lung-resident dendritic cells bridge the innate and adaptive immune response through antigen presentation. Activated local innate immune cells produce cytokines and chemokines that amplify the inflammatory response, including activation of fibroblast units [18, 35, 51, 52], and together stimulate the infiltration of immune cells such as monocytes, neutrophils, and lymphocytes into the lung. SP-A and SP-D contribute to these responses, modulating the function of macrophages, dendritic cells, neutrophils, and T cells, modulating their level of inflammation [18, 64]. Neutrophils contribute to resolution of infection by engulfing microbes, by producing antimicrobial peptides such as the protease cathepsin G, and by creating neutrophil extracellular traps (NETs) in a process known as NETosis. NETs are mesh-like structures containing DNA fibers, histones, cytotoxic proteins, and proteases, which are expelled into the extracellular space by activated neutrophils, capture microbes, and keep them from entering and damaging cells [65].

During the resolution of acute infection, return to homeostasis from inflammatory stressors depends on inactivation of the immune system and on the fibroblast units’ efficacy for resolving the assault (e.g., wound resolution) [18, 35]. This process requires the removal of acute stimuli (e.g. residual pathogen or toxic agent), eliminating triggers for activation of innate and adaptive immune cells. Some studies have suggested that this is an active process which involves altered metabolism of polyunsaturated fatty acids (PUFAs), leading to production of inflammation antagonists known as specialized pro-resolving mediators (SPMs) (e.g., lipoxins, resolvins, protectins, and maresins) [66], which limit collateral injury to healthy tissue [66–70]. Biological functions attributed to SPMs include elevation of phagocytosis and efferocytosis, increased cytotoxic cell killing by NK cells, and elevated Treg responses and IL-10 production, and decreased activity of the Th1 and Th17 pro-inflammatory T cells (which produce proinflammatory cytokines IL-17, IL-21, and IL-22), reduced production of other inflammatory cytokines, and reduced inflammasome formation [71]. Mechanistically, to produce these effects, SPMs bind to G-protein coupled receptors (GPCRs) and trigger signaling cascades that inactivate NF-кb and other downstream pro-inflammatory effectors [72]. However, the relative importance of SPM function versus other mechanisms requires considerable additional investigation, given some data which argues against the importance of this mechanism [73].

Following tissue insult, there is dynamic reciprocity between the epithelial, endothelial, mesenchymal, and immune compartments, which communicate with each other to repair injury, but can also engage in a feed-forward cycle that intensifies pathological responses [74, 75]. These interactions are now appreciated as governing the dormancy versus awakening of DTCs.

## Seeding of dormant DTCs in the lung

**Fig. 2:**
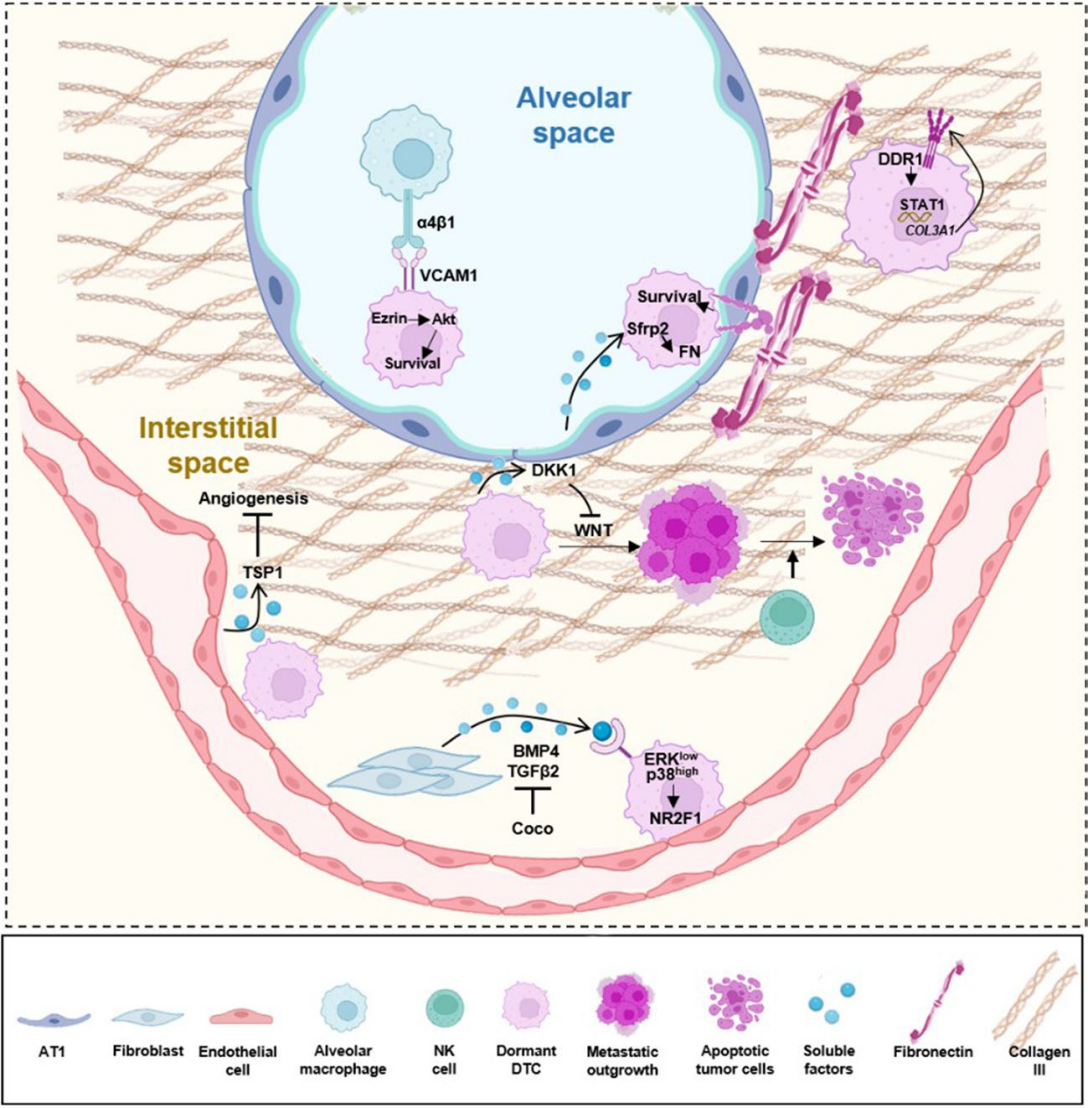
Signaling supporting DTC dormancy in the lung. Cues from the lung microenvironment are critical in maintaining dormancy of DTCs. In the alveolar space, DTCs expressing VCAM-1 receive survival signaling from resident macrophages through juxtacrine activation of a VCAM-1-Ezrin-PI3K/Akt survival pathway. In an epithelial niche, induction of SFRP2 in DTCs causes them to conduct de novo fibrillogenesis, resulting in increases in fibrillar fibronectin as a component of the ECM being altered, and thus promoting survival signaling. Enrichment of type III collagen in the interstitial ECM niche also sustains DTC dormancy, signaling through the DDR1 receptor to activate STAT1. In the perivascular niche, deposited basement membrane components like TSP-1 suppress angiogenesis to also support dormancy. Local resident fibroblast/ECM units secrete TGFβ2 and BMP4 to inhibit ERK1/2 signaling, promoting dormancy. Additionally, DTCs can enter dormancy by secreting DKK1, an inhibitor of the WNT signaling pathway, and evading NK-cell-mediated immunity within the dormant/metastatic niche. See text for details. This figure was created in part using BioRender (BioRender.com)

Dormancy is typically induced in the vicinity of the primary tumor mass before cancer cells disseminate from the primary site and help protect cells as they transit through the circulatory system [3]. Numerous signaling mechanisms have been identified that contribute to induction of dormancy in DTCs. These have been extensively and recently reviewed (e.g., [76, 77], among others), and the mechanisms identified to induce dormancy in this large and growing field will not be presented here in detail; Fig. 2 summarizes key factors and pathways known to induce dormancy in the lung.

The local microenvironment of a dormant DTC may vary based on point of arrival [78, 79]. DTCs disseminate by numerous routes (hematogenous, lymphatic, or diffusion through the pleural cavity) [80, 81]. To date, the majority of studies of dormant DTC awakening focus on cells that have arrived via a hematogenous route, in part because many studies performed in mouse models rely on intravenous injection of DTC models [80, 82]. DTCs arriving via the hematogenous circulatory system typically enter the lung after becoming lodged in the capillary network. This lodges them in proximity to the alveolar cells. DTCs diffusing through the pleural cavity will lodge in the visceral pleura. The point of lung entry for DTCs initially traveling through the lymphatic system has not been as well studied [80, 81]. Mechanistically, one fascinating recent study has identified a role for periostin expressed by cancer associated fibroblasts (CAFs) in the primary tumor as having a key role in producing an extracellular track that invasive DTCs can use to enter the lymphatic system [83]**.** Such DTCs may ultimate enter the lung by the hematogenous system, as lymphatics drain directly through the thoracic duct or right lymphatic duct into the subclavian vein [84]; in addition, if DTCs pass through lymph nodes during transit, this is potentially another site of transfer to the hematogenous vascular system [85]. In this case, they would end up trapped in capillaries, proximal to alveoli. Alternatively, these cells may evade lymph nodes and remain in the lymphatic system [85] and enter the thoracic duct and collecting vessels against the flow of lymphatic drainage, allowing them to lodge more broadly throughout the lung [86]**.** There is some evidence supporting the importance of this route, including the observation that identified specific associations between higher density of lymphatics with metastases and poor outcomes [87], as well as striking findings that melanoma DTCs transiting through lymph nodes are protected against ferroptosis, whereas those solely transiting through blood vessels are not [88].

When DTCs arrive in the lung, their arrival is typically preceded by the establishment of a niche that allows them to survive in the new environment. The pre-metastatic niche (PMN) has been most studied, reflecting the fact that many DTCs arriving in these niches soon begin proliferation into overt metastases. The features of PMNs have been thoroughly reviewed [89–94], and detailed discussion of the PMN is beyond the scope of this article, but knowledge of key elements of a PMN provides a useful contrast to the features of a pro-dormancy niche. Briefly, establishment of PMNs is induced by signals sent out from the primary tumor, which affect the stromal tissue and alter the local fibroblast units, including dynamic alterations to their ECM that result in release of factors stored in the ECM (e.g., ligands of the TGFβ family), and immune cells to enhance DTC survival and growth into a metastasis [18, 95, 96]. Critical signals include the release of extracellular vesicles (EVs) such as exosomes that bind to macrophages, and other cells, in the target organ to induce immunosuppression [90]**.**

Numerous mechanisms by which tumor-produced EVs support metastasis formation have been described. EVs binding TLRs on AT2 cells of the lung epithelium cause them to release chemokines and metabolites that recruit neutrophils [97, 98]. Recruited, activated neutrophils produce NETs; components of the NETs, including extracellular DNA [99], help recruit DTCs to the pre-metastatic niche. Cathepsin C produced by primary breast tumor cells also contributes to NET formation [100]. Interaction of breast DTCs with capillaries cause the vascular cells to produce tenascin-C, which interacts with TLR4 on macrophages, producing nitrous oxide and tumor necrosis factor (TNF), further stimulating production of components required for a PMN [101]. In the lung, an organ-specific repository of metabolites such as the fatty acid palmitate contributes to increased production of acetyl-CoA in breast DTCs, supporting epigenetic reprogramming of NF-κB in a way that promotes metastatic growth [15]. Breast DTCs also secrete factors including IL-1α and IL-1β to activate signaling in lung fibroblasts, triggering these cells to produce cytokines that create a pro-inflammatory, pro-growth environment [102]. EVs bind Toll-like receptors (TLRs) on macrophages to elevate expression of immune checkpoint proteins such as PD-L1 [103]. Numerous components of the ECM, including specific collagens, glycoproteins, and proteoglycans, have also been associated with metastatic outgrowth [104]. Together, this interchange of signals between DTC, fibroblast, immune cell, endothelial cell, and ECM allows the rapid growth of metastases from DTCs.

## Mechanisms for maintaining DTC dormancy, and features of dormant DTCs

In contrast to behavior of DTCs in a PMN, many DTCs remain dormant (as single cells), or form small micro-metastases and cease growth upon arising at a site distant from the primary tumor. For this reason, there is thought to be diversity in the characteristics of the niches that receive DTCs, with some promoting dormancy rather than promoting growth. Insofar as it is more difficult to track the behavior of individual non-proliferating cells, the features of niches supporting dormancy are less well explored. However, the role of a perivascular niche (PVN), adjacent to the capillary basement membrane, is now well established. Roles have also been proposed for an *ad hoc* niche [105], produced by signals from arriving DTCs that condition the local fibroblastic stroma and immune cells, and the native stem cell niche, representing pre-existing cellular compartments in which stem cells for the tissue reside (reviewed in [89, 106]). The role and importance of these latter niches may have distinct features in different tissues [89, 107].

In the identification of dormant DTCs after they have established in a distant site, consistent features emerge. At the most level, dormant breast and prostate DTCs do not express proliferative markers such as Ki-67 and PCNA and upregulation of cyclin dependent kinase (CDK) inhibitory proteins including p21 and p27 [108]. One key factor is the reprogramming of cells to express a program of transcription factors associated with stem cell and dormancy phenotype, including SOX9, SOX2, RARβ, NR2F1, and other factors (ZFP281, FOXA1, GATA3 [3, 109]**.** Another hallmark is reprogramming of autocrine and cytoplasmic signaling cascades away from a pro-proliferative state to a state similar to quiescence. For example, autocrine expression of the WNT pathway inhibitor Dickkopf-1 (DKK1) reduces WNT signaling; TGF-β signaling is enhanced; the stress-associated kinase p38 is more active; and the proliferation-associated kinase ERK1/2 is less active [110–112]*.* Activation of p38 stress responses triggers the unfolded protein response (UPR) [113], together with action of the NRF2 transcription factor, which reprograms redox and nucleotide metabolism; these changes make dormant cells more resistant to stress and nutrient limitation [114–116].

Collectively, changes that maintain dormancy are induced by physical interaction of a DTC with cells in its microenvironment or receipt of soluble signals produced by these cells (Fig. 2). Critical pro-dormancy interactions occur with endothelial cells, fibroblast/ECM units (that is, the specific ECM associated with distinct types of fibroblast), macrophages, and other immune cells and are mediated by cell surface receptors including VCAM-1, TGFβ family receptors, integrins, and others [3, 117]. Specifically in the lung, several features of the PVN have been defined as important for promoting dormancy. A study of breast cancer metastasis to the lung determined that dormant DTCs lodged in established microvasculature. This local microenvironment included upregulated thrombospondin 1 (TSP1), a component of the ECM that is anti-angiogenic and proapoptotic and actively inhibited breast DTC awakening [107]. Importantly, neovascular formation following tissue damage reduced TSP1 expression and induced lung metastatic outgrowth.

A second key factor is bone morphogenetic protein 4 (BMP4), a member of the TGFβ superfamily, which induce activation of NR2F1, a master regulator of dormancy [118, 119]. BMP-mediated suppression is antagonized by COCO (also known as DAN domain BMP antagonist family member 5 (DAND5)), a secreted antagonist of TGFβ ligands. COCO blocking of paracrine BMP signaling enhances the self-renewal capability of metastasis-initiating cells [118]. BMP signaling is also antagonized by GALNT14 which has elevated levels of expression in lung-metastatic breast cancer cells [120] and LAPTM5, a lysosomal transmembrane protein, which degrades the critical BMP receptor BMPR1A [121]. How the activity of LAPTM5, COCO, and GALNT14 is restrained during maintenance of dormancy requires further study.

An interesting study has shown that the interaction between AT1 cells and DTCs causes the latter to produce the WNT antagonist secreted frizzled-related protein 2 (sFRP2); sFRP2, in turn, promotes *de novo* local fibronectin fibrillogenesis, causing local integrin-dependent pro-survival signals that allow maintenance of the dormant breast DTC [122]. Another group has used co-culture experiments to show that interaction with AT1 cells cause DTCs to produce ephrin B6 (EPHB6), contributing to their survival [123].

There has long been substantial evidence for altered gene expression and signaling in dormant DTCs that promotes immune system evasion, based on studies performed in various tissues. Dormant DTCs downregulate epitopes including the MHC-1 proteins and required to stimulate anti-tumor T and NK cell activity [111, 124–126]. Mechanistically, a contributing factor to these gene expression changes and escape from T cell killing appears to be a partial blockade of the endoplasmic reticulum (ER) stress response, in which a protective stress response is apparent based on transcriptomic signature and activation of the PERK pathway, but unresolved due to lack of expression of IRE1a and XBP [126]. In addition, DKK1 activation downregulates a group of UL16-binding proteins (ULBPs) that are ligands that bind the NK receptors NKG2D/CD314 and activate NK cells, thereby shielding the dormant DTC from NK-induced cell killing [111].

The stimulator of interferon genes (STING) pathway, which is considered to be the main source of the production of type I interferon in the tumor microenvironment, also prevents the progression from the indolent state [127]. Dormant cells are also more dependent on autophagic pathways, which extend the ability of dormant cells to survive in a stressed, nutrient-restricted state [128, 129]**.** One study of dormancy induced by nutrient limitation of established breast cancer models described substantial divergence among cell models, with those cell models capable of long-term survival producing heterogeneous colonies containing quiescent, proliferating, and senescent cells. However, a common feature of all models included the ability to produce a fibrillar fibronectin matrix (dependent on α_v_β_3_ and α_5_β_1_ integrin activation of ROCK-generated tension) and autocrine production of TGFβ2 [130]. One recent study has also demonstrated that dormant tumor cells condition the ECM to produce a niche that is rich in type III collagen, which limits Discoidin Domain Receptor Tyrosine Kinase (DDR1)-dependent STAT1 activation [105], restricting awakening.

In considering mechanisms regulating dormancy maintenance, several issues should be kept in mind. First, it is becoming well appreciated that the tumor microenvironment varies considerably among tumors [131]. Hence, there is likely to be considerable cell type and tumor type-specificity to the maintenance of dormancy. While dormancy has been extensively investigated for some tumor types, for others little is known. Second, as cells migrate from the primary tumor, some migrate as single cells, whereas others utilize collective migration [132–136]; there is evidence that cells migrating as clusters are more effective at seeding metastases, for at least some tumor types [136, 137]. Whether this reflects a change in dormancy initiation, dormancy maintenance, or other pro-metastatic processes is not known. Third, this field is actively evolving, and much remains unknown. For instance, the recent discovery of a role for intratumoral bacteria of the primary tumor mass in conditioning DTCs for survival during dissemination [138, 139] and the recognition that there are inherited predisposition to relevant lung pathologies, such as pulmonary fibrosis [140], open entirely new areas for investigation. Emerging literature suggests sex differences in some of these pathogenic conditions [141], adding another level of complexity.

## Awakening of dormant DTCs and metastatic outgrowth in the lung: consequences of cancer treatments

Lethality of cancer is often associated with the awakening of DTCs in the lung. Both tumor growth and systemwide responses to cancer treatments are linked to inflammatory processes (reviewed in [142]). Inflammation is a complex process that can both be tumor-promoting and tumor inhibitory based on type of cancer and type of therapy applied [143, 144]. However, both inflammation and modifications of ECM in the TME associated with treatment-induced and other sources of fibrosis are strongly linked to tumor progression as well as DTC awakening [18, 35, 53]. In the lung, inflammation, fibrosis, and ECM changes are often interdependent and can arise from multiple sources, including ancestry; lifestyle choices such as tobacco smoking; the aging process itself; or paradoxically, response to treatments intended to control the primary tumor.

**Fig. 3:**
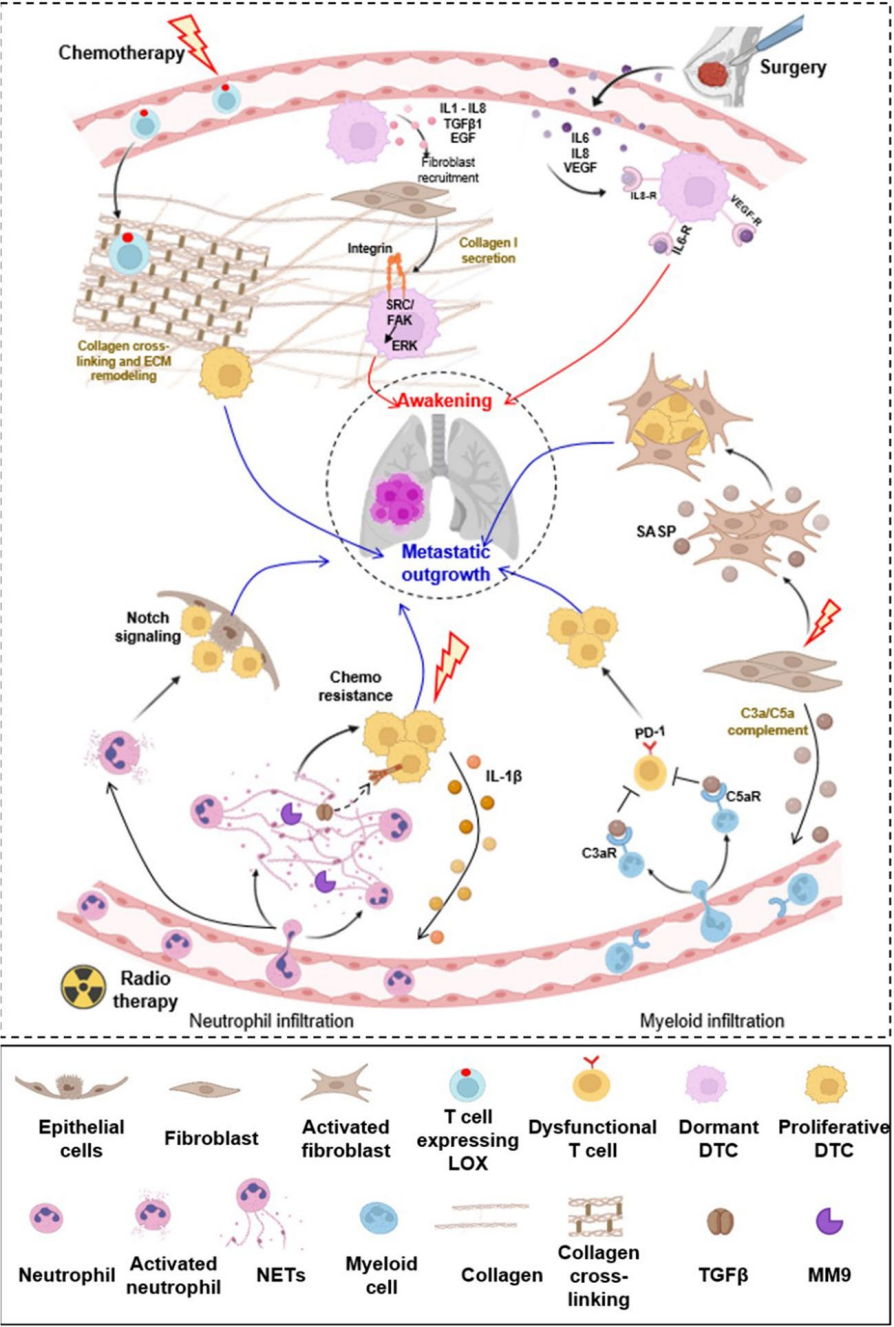
Signaling activated by cancer treatments that promote awakening and metastatic outgrowth. Chemotherapy promotes infiltration of LOX-secreting CD8^+^ T cells in the lung, which modulates the ECM, promoting metastasis. Chemotherapy also stimulates tumor cells to release cytokines including IL1β, IL8/CXCL8, TGFβ1, and EGF, that caused fibroblast recruitment. Fibroblasts then secrete collagen-1 and COX2, triggering integrin/SRC and prostaglandin E signaling in the cancer cells, stimulating awakening of dormant DTCs. Chemotherapy action on lung DTCs stimulates them to secrete IL-1β, which in turn triggers NET formation, promoting chemoresistance and promoting metastasis. Furthermore, chemotherapy promotes complement signaling in lung fibroblasts, leading to recruitment of MDSCs and the formation of an immunosuppressive niche, favorable to metastatic relapse. Other pro-metastatic mechanisms linked to cancer treatment include the release of pro-inflammatory cytokines and growth factors such as IL-6, IL-8, and VEGF, which awaken DTCs and micrometastases in the lung. Additionally, radiation in healthy lung tissue leads to infiltration and activation of neutrophils and induces Notch activation within epithelial cells, fueling the subsequent growth of arriving DTCs. Additional details are provided in the main text. This figure was created in part using BioRender (BioRender.com)

Chemotherapies induce numerous changes in signaling pathways [145, 146] that can affect both DTCs and all cells that interact with it, leading to a paradoxical promotion of metastasis following treatment. Figure 3 summarizes the key factors and pathways known to induce awakening and metastatic outgrowth in the lung after cancer treatments (surgery, radiotherapy, and chemotherapy). We note that it is likely that the complexity and heterogeneity of this process are greatly underappreciated at this time, as the application of single cell sequencing techniques is revealing a diverse set of pathways recapitulating elements of normal tissue development as DTCs begin to form metastases [147].

ECM abnormalities linked to the formation of fibrosis are common sequelae to cancer treatments [18, 35]. Under conditions of treatment-induced acute or chronic inflammation, pericytes differentiate into scar-forming myofibroblasts, while local resident fibroblast units are activated to simulate wound healing. This leads to dynamic changes in the interstitial ECM, including increased collagen deposition, matrix stiffening, and other changes that drive and sustain tissue fibrosis [18, 35, 50]. Besides signaling changes discussed below, chemoradiation can induce cellular senescence, with senescent cells participating in dynamic changes to the local ECM in a manner that also promotes fibrosis [148–151]. Lung fibrosis is associated with radiation, chemotherapy, and surgery.

Radiation-induced lung injury (RILI) which occurs in up to ~ 20% of treated patients is marked by an early phase of radiation pneumonitis (RP), which occurs 2 to 6 months after radiation therapy and is characterized by acute lung tissue inflammation. Early changes induced by RILI/RP include oxidative stress pathway activation in AT2 cells and extensive immune cell infiltration [152]. In addition, at a later phase, RILI causes fibrosis resulting from chronic tissue damage; this can occur years after treatment [153]. Factors mediating the pathologic effects of RILI include activity of the inflammatory cytokine TNF-alpha [154] and specific changes in tricarboxylic acid (TCA) cycle energy metabolism that provide increased energy availability for fibroblast proliferation [155]. RILI also activates the cGAS-STING signaling pathway—a potent inducer of innate immune and subsequently adaptive immune responses, associated with the recruitment and the polarization of macrophages which contribute to the establishment of a pro-inflammatory and pro-fibrotic environment [156]. Several chemotherapy drugs can also induce lung damage (sometimes termed drug-induced interstitial lung disease (DIILD) or chemotherapy induce pulmonary toxicity (CIPT)) in a subset of treated patients [157, 158]. Early responses include elevated local production of TGFβ and inflammatory cytokines and enhanced neutrophil infiltration; longer term effects can include extensive fibrosis [159].

A recent multiomic analysis of a dormancy breast cancer cell model co-cultured *in vitro* with endothelial cells and stromal fibroblasts in a 3D setting found that docetaxel-damaged fibroblasts secrete IL-6, G-CSF, MIP-2, and TNFα, promoting awakening of dormant tumor cells [39]. Antibodies blocking IL-6 and/or G-CSF prevented awakening from dormancy for the breast cancer cells*.* *In vitro* and *in vivo*, cancer cell awakening was promoted by activation of the MEK kinase; interestingly, after initial awakening, cancer cells also initiated autocrine IL-6 stimulation, preventing return to dormancy. Systemic chemotherapy treatments also enhance metastatic outgrowth in the lung by inducing a senescence-associated secretory phenotype (SASP) expression in normal lung fibroblasts [160]. Other studies have also demonstrated that treatment of mesenchymal cells with either doxorubicin or paclitaxel alters their secretion of cytokines such as IL-8 and conditions them to support growth of breast cancer cells [161]. While some taxane-based drugs such as eribulin can stimulate TGFβ inhibition to potentially prevent DTC awakening [162, 163], paclitaxel activates CD8 + T cells to promote expression of the enzyme lysyl oxidase (LOX), causing ECM remodeling and changes in the stiffness of the lungs and creating a fibrotic environment that promotes metastatic outgrowth [164]. These studies suggest that the choice of chemotherapeutic agents could protect from or enhance metastatic DTC awakening in the lung.

Mechanistically, the induction of fibrosis in lungs is promoted by inflammatory cytokines, by factors such as TGFβ, which increase expression of collagen-1 and other key ECM components, and by factors such as proteases and LOX, which dynamically alter collagen bundling and the biomechanical composition of the ECM [51]. One interesting study has shown that treatment with paclitaxel + cisplatin caused lung tumor cells in the lungs to activate the transcription factor ATF6, stimulating the release of cytokines including IL1β, IL8/CXCL8, TGFβ1, and EGF, which caused fibroblast activation and their recruitment to the tumor site. These CAFs initiated a forward “vicious cycle” of microenvironmental field expansion (akin to fibrosis) that triggered pro-tumoral ECM changes. These changes included augmenting collagen-1 and COX2 production and a reciprocal signaling induction of cancer cell integrin/SRC and prostaglandin E signaling that stimulated metastatic awakening in the lung [165]. An independent study also found that collagen-1 provides ligands for dormant DTCs expressing integrins, directly activating known cell-ECM adhesion-responding kinases, such as SRC and FAK, resulting in the downstream activation of ERK to promote DTC awakening in lung cancer [166].

In addition, collagen signals through its receptor DDR1 and the DDR1-associated tetraspanin TM4SF1 to activate a signaling cascade through PKCα and STAT1/JAK2 that triggers SOX2 and NANOG expression, inducing metastatic outgrowth of dormant breast cells [167]. Crosslinked collagen also signals through integrins to activate PI3K, providing a separate stimulation to growth and invasion of tumor cells [168]. Reciprocally, MMP8 and MMP9-induced dynamic ECM alterations to collagen and other matrix components (such as elastin) during the inflammatory process result in the production of “matrikines” (e.g., small ECM peptides) such as N-acetyl Pro-Gly-Pro (Ac-PGP) [169]. Ac-PGP is known to act as a potent recruiter of neutrophils, creating a feed-forward system to ensure continuing tissue damage [169]. Ac-PGP is known to increase the recruitment of tumor cells to the site of tissue damage [170]; although an effect on awakening is not known, it is likely given the defined biological activities of this peptide. Another well studied matrix metalloprotease, MMP2, has specifically been shown to contribute to awakening from dormancy via induction of *de novo* fibrillogenesis by the fibroblastic/ECM unit through degradation and remodeling of the interstitial fibronectin fibers, which unleashes a dormancy-promoting cascade event [130].

Interestingly, surgery has also been linked to both early and late metastatic recurrence [171, 172]. Although the link between surgery and metastasis is typically thought of as contributing to the mechanical dissemination of cancer cells shed from the tumor at the time of its removal, surgery at any site can stimulate inflammation, surgery-associated immunosuppression, neovascularization and activation of coagulation factors, and tissue rearrangement that can promote the awakening of dormant tumor cells. While inflammation is localized to the site of the surgery, systemic effects also occur that over the long-term result in outgrowth of dormant DTCs at distant sites [173, 174]. Acute responses to surgery include release of pro-inflammatory cytokines such as TNFα, IL1β, IL-6, and IL-8; longer-term responses include release of TGFβ [173]. One study using mouse models has suggested that surgical manipulation at distant sites activates systemic β-adrenergic and prostaglandin signaling, causing cells derived from a primary tumor to secrete IL-6, IL-8, and VEGF, activating growth of micrometastases [175]. Similarly, surgery at distant sites can induce LOX activity in the lung, conditioning tissue remodeling and elevated collagen deposition, linked to activation of integrin-dependent cell matrix adhesion-induced signaling and metastasis in the lung [174].

Cancer patients often have tissue damage that leaves them vulnerable to infections [176]. In a mouse model of breast cancer, chronic pulmonary bacterial infection was found to facilitates lung metastasis by recruiting tumor-promoting MHCII^hi^ neutrophils [177]. Stress-activated neutrophils forming NETs directly contribute to awakening in a number of ways, including by releasing pro-inflammatory damage-associated molecular patterns (DAMPs), the S100A8/A9 complexes [178]. These DAMPs stimulate myeloperoxidase, elevate oxidize lipids, and thereby induce a fibroblast growth factor receptor (FGFR) pathway in dormant lung tumor cells to stimulate outgrowth of metastases [179]. Intriguingly, besides promoting initial awakening, some work has suggested neutrophils contribute to successful outgrowth of dormant tumor cells in additional ways, including promotion of chemoresistance (e.g., [180]) and by provision of nutrient reserves to growing metastases in breast cancer [181]. In addition, NETs are present in the blood of cancer patients [182, 183], and studies in mouse models indicate NETs can be induced by cancer cells in the lung vasculature to promote extravasation and invasion [99, 184].

In a key study, Albrengues and colleagues demonstrated that NETs also directly activate dormant tumor cells. In this study, the authors induced sustained lung inflammation in a mouse model of dormant breast cancer, either by exposing mice to cigarette smoke extract or by nasal instillation of bacteria-derived liposaccharide. This produces NETs in the vicinity of the dormant tumor cells; these NETs encompass the secreted proteases elastase and MMP9, which cleave the ECM protein laminin to release a known matrikine peptide that can activate integrin α3β1 on the surface of dormant tumor cells. Activation of this integrin triggers downstream signaling through a FAK/ERK/MLCK/YAP signaling cascade and converts dormant cells to aggressive metastases [185]. Subsequent work demonstrated that cisplatin induced breast tumor cells seeding the lung to secrete CXCL1 and CXCL5, leading to neutrophil recruitment, and IL1β, causing NET formation [180]. Other studies have found that irradiation of a healthy lung in a mouse model causes infiltration of neutrophils and subsequently enhanced DTCs colonization and metastases [186]. Finally, in a real-world setting, these activities of neutrophils may be influenced by patient-specific lifestyle settings such as obesity, which has been shown to influence neutrophil activation, NETs, and breast cancer metastasis to the lung [33, 187].

Following treatment with chemotherapy or radiation, cancer patients often have some degree of immunosuppression due to depletion of lymphocytes [188] and systemic increases in TGFβ [18, 35]. This leads to loss of immunosurveillance, enhancing the growth of micrometastases. For example, one detailed profile of the consequences of doxorubicin versus cisplatin on metastasis in a mouse breast cancer model showed that only doxorubicin caused lung CAFs to secrete complement system components; these caused recruitment of myeloid-derived suppressor cells (MDSCs) to the lung, quenching local T cell responses and contributing to metastatic relapse [38]. In related findings, gemcitabine was also found to induce MDSC accumulation in the lung [189], while paclitaxel and doxorubicin treatment elevated CCL2 expression in the lung, causing recruitment of CCR2-expressing, bone marrow-derived macrophages; these cells in turn produced coagulation factors such as activated FX and induced a pro-metastatic effect [190]. Such results emphasize the importance of better methods for determination of whether patients with surgically removed early stage tumors additionally treated with chemotherapy are likely to have shed micrometastases or not.

Many patients with invasive tumors that have spread to lymph nodes but have no overt metastases are treated with targeted therapies or immunotherapies. Whether and how these agents influence the likelihood of metastatic recurrence is a topic of high clinical interest. However, particularly for immunotherapies, there is limited information available, in part because there is not a long track record of use of these drugs, limiting development of suitable cohorts for comparison. Among the older targeted therapies, a number of those targeting signaling proteins such as EGFR, HER2, KRAS, KEAP, STK11 and mTOR, or cell cycle regulators such as CDK4/6 or WEE1 have now been recognized as having substantial effect in modulating the immune system (e.g., [191–196]). Activities induced by inhibitors of these targets include pro-inflammatory cytokine production, downregulation of the immune checkpoint protein PD-L1, recruitment and or activation of NK cells and macrophages, depletion of MDSCs, and reduced expression of immunosuppressive factors [197].

Based on studies summarized above, these pro-inflammatory signals may hence act on lung tissue so as to enhance tumor awakening, by promoting fibrosis and local inflammation, and some evidence in the scientific literature supports this interpretation for some targeted inhibitors (e.g., [198, 199]). PD-L1 is expressed on cancer cells, CAFs, Tregs, MDSCs, and macrophages and PD-1 on T cells and NK cells; immunotherapies targeting PD-L1 and PD-1 will have broad effects in the microenvironment of the lung, and immunotherapy-related lung injury (IRLI) has emerged as a topic of concern [200]. Notably, immunotherapies are known to work best in an “immune hot” or inflamed rather than an “immune cold” environment, and various approaches are being employed to elevate inflammation to increase anti-tumor responses and decrease immune tolerance, including combination of targeted drugs with immune checkpoint inhibitors [197]. It seems quite plausible that such pro-inflammatory treatments will influence dormancy and increase awakening, although this remains to be determined. Whether such awakening is beneficial for treatment in mobilizing dormant cells to an active growth condition in which they are killed, or counter-productive in increasing the number of metastatic outgrowths that evade treatment, remains to be determined.

## Strategies to prevent treatment-mediated awakening

There is a rich literature on the development of therapeutic strategies to reduce metastasis ([145, 201, 202], and many others). The majority of these strategies address treatments administered at the time of surgery or chemoradiation to treat the primary tumor, reflecting the fact that these damaging treatments can increase the dispersal of tumor cells from its site of formation [203]. For this purpose, strategies to limit extravasation, survival in the circulatory system, immune evasion, and intravasation all make valuable contributions. In addition, some approaches focus on trying to limit the formation of a PMN; for example, using a nanoparticle in which the chemotherapy doxorubicin is enclosed in a micelle containing all-trans retinoic acid (ATRA), to reduce NF-кB signaling and deplete MDSC recruitment and expressing hyaluronic acid to increase targeting to the site of cancer cells [204]**.** These strategies typically seek to limit metastases by interventions at the time of initial therapeutic treatment. In contrast, strategies to specifically target the process of awakening must take into account the fact that this process can occur within a few months to many years after initial treatment. Hence, while some strategies to eliminate dormant tumor cells may address the acute pro-metastatic stimuli induced by treatment of the primary tumor, it is also important to consider strategies that can be used for years after initial cancer treatment as means for intercepting metastatic DTC awakening.

Among anti-awakening therapies that can be administered at the time of treatment to block processes that reverse dormancy, control of inflammation (including stimulation of inflammatory resolution [205, 206] is central. An example of such therapy would be the use of drugs that reduce formation of fibrotic tissue in the lung induced by chemoradiation, targeted, or immunotherapy. Provocative evidence from a number of recent preclinical studies suggest that inflammation and inflammation resolution can be manipulated to reduce awakening and eradicate micro-metastases [127, 179, 207, 208]. For example, one preclinical study has shown that use of inhibitory antibodies or small molecule agents to block IL-6, G-CSF, or MEK/ERK—components of a key inflammation-associated awakening pathway—prior to the administration of the chemotherapy docetaxel, was effective in reducing emergence of metastasis [39]. Other approaches used to control inflammation include treatment with STING agonists [127]; presurgical administration of specific non-steroidal anti-inflammatories or SPMs [207]; or inhibition of β2-adrenergic receptors with the β-blocker ICI-118,551 to inhibit release of the pro-inflammatory S100A8/A9 factors, or with an FGFR inhibitor to block a key autocrine loop in awakening tumor cells [179]. It has been suggested that strategies to increase the rate of clearance of tumor cells killed by chemotherapy, potentially by introduction of resolvins, may limit pro-tumoral inflammation [209]. Based on numerous roles of NETosis in promoting tumor growth, this process has also been suggested as a potential target, with multiple approaches to inhibit this process outlined in [210]. Notably, inhibition of PAD4, a NETosis trigger, has been shown to reduce metastatic spread for ovarian cancer [211], while other strategies have reduced the early appearance of biomarkers of metastasis [212]. A number of pain relieving drugs, including non-steroidal anti-inflammatory drugs (NSAIDs), have immunomodulatory effects mediated by inhibition of prostaglandin signaling [213], and their level of use during and subsequent to cancer treatment may influence the behavior of dormant tumor cells. The use of senolytic drugs to remove treatment-induced senescent cells may also be efficacious, as these limit lung fibrosis [151].

Besides employment at the time of initial cancer treatment, treatments to suppress inflammation may be useful over the long term in eliminating metastatic recurrence. In addition, with the growing identification of molecular mechanisms responsible for promoting awakening, it is possible to modulate a number of pro-dormancy signaling pathways with targeted inhibitors. For example, preclinical studies have shown that treatment with an inhibitor of lysophosphatidic acid receptor 1 (LPA1) elevates p38 and reduces ERK activation, promoting dormancy of breast cancer cells [214]. Combination of the epigenetic modulator 5-azacitidine with ATRA was able to enhance an NR2F1-dependent transcriptional profile [109].

Multiple studies have explored inhibition of cell-ECM signaling by targeting integrins and/or its effectors (e.g., SRC, ERK, FAK), given the importance of signaling by fibrous collagen (e.g., collagen I, III) as well as additional ECM components induced during therapy-induced fibrosis [166]. Using an alternative receptor, DDR1, collagen activates JAK/STAT signaling during tumor awakening [167]; JAK/STAT inhibitors can reverse this program. Given the observation that COCO and other factors awaken cells by inhibiting BMP4 activity [118], agents targeting the BMP/TGFβ signaling pathway may also have value in limiting metastatic recurrence. It may also be possible to target the PVN, rendering DTCs vulnerable to some forms of therapy [215]. In this regard, supporting expression of TSP1 and reducing expression of factors associated with neovascularization, such as periostin and tenascin-C [216], would reduce awakening. Glucocorticoid receptor activation has been shown to induce a dormancy-like state. Intriguingly, while this is accompanied by reduced sensitivity to many chemotherapies, it is associated with a substantial sensitization to inhibitors of IGF-1R [217]. Similarly, targeting of DKK3—produced by dormant tumor cells to impede immune clearance—may help eliminate DTCs while still in the dormant state [218]. Inhibition of PERK, and resulting impairment of integrated stress survival signaling, has also been proposed as a method of reducing viability of dormant DTCs and hence suppressing metastatic recurrence [219].

These approaches have shown promise in the preclinical setting. However, at present, there is little clinical evidence to indicate the feasibility of maintaining patients on such treatments over the longer term. Whether chronic use of pathway-targeted therapies is sustainable will depend on numerous factors. These include the side-effect profiles of the drug employed; the cost of the therapy; and the risk that the treatment raises the likelihood of adverse events unrelated to cancer recurrence. For example, while there is evidence that some sources of chronic inflammation are associated with cancer recurrence, immunosuppression is also associated with numerous risks [220, 221]. At present, the greatest promise is evident for treatments used within weeks to months of cancer treatment.

## Emerging issues, future directions: lifestyle and aging

Many key questions about the relation between cancer treatment and DTC awakening remain unaddressed. In particular, as basic mechanisms governing awakening become established, how these factors are regulated in a real-world setting has attracted growing awareness. Areas of particular interest include patient lifestyle and patient aging.

Among the lifestyle factors that can influence metastatic recurrence in the lung of any individual patient, a number of studies have implicated smoking, for example, the expression of the matrikine Ac-PGP, as this peptide is elevated as consequence of exposure to tobacco smoke [70, 222]. In addition, NET formation has been shown to be induced by nicotine and its main metabolite, cotinine [223, 224], and NET abundance is elevated in smokers compared to non-smokers [224]. Overall, the condition of the lung in smokers is chronically inflamed, with up to 15% of smokers develop chronic obstructive pulmonary disease (COPD), causing a distinct signaling landscape [225, 226]. Individuals with COPD are often treated with systemic glucocorticoids to improve lung function and alleviate symptoms [227]; this may condition the microenvironment of DTCs to further accelerate awakening following cancer treatment [217]. Arguing in support of this idea, an intriguing recent study has found that chronic stress contributes to metastasis formation by triggering glucocorticoid release and thereby enhancing NET formation, causing a metastasis-promoting microenvironment [228].

Recent studies have suggested that other sources of lifestyle-lined inflammation, such as obesity, can also influence formation of pulmonary metastases, in line with a growing literature about the relationship between obesity, diet, and inflammation [32, 229–232]. In addition, specific components of diet have been identified as influencing immune cell functions [233]. High polyphenol intake might reduce breast cancer recurrence: Vegetables, fruit, fish, and whole grain consumption have been linked to reduced inflammation and the Mediterranean diet shown to reduce the levels of proinflammatory markers [234]. Conversely, dietary intake leading to metabolism of arachidonic acid by cyclooxygenase and lipoxygenase pathways produces metabolites that could be involved in dormant cell reactivation [235]. The recent success of immune checkpoint inhibitors in obese cancer patients has raised the possibility that additional immune-targeted therapies may hold therapeutic value in restraining metastatic recurrence [231].

Most cancers are rare in individuals under 40 years of age, with incidence significantly elevated as individuals age into their 50 s and beyond. As a result, metastatic recurrence is also most common in aged individuals. There is growing recognition that older age conditions the lung microenvironment in many ways that can influence dormancy versus awakening. Some work has suggested that the epithelial and mesenchymal cells of the lung may be uniquely poised for cellular senescence, based on their exposure to high oxygen and environmental challenges [151]. Older age is a common risk factor for damage to the lung following use of antineoplastic agents [236]. Preclinical studies using mouse models have demonstrated that the elevation of expression of PDGF-C in aged or fibrotic lungs in each case contributed to enhanced awakening and metastatic relapse of estrogen receptor-positive breast cancer cells [237]. There is substantial interaction between the metabolic changes that occur during aging and those that characterize cancer cells [238]. As one example, age-induced accumulation of methylmalonic acid (MMA) can cause production of proteins that remodel cytokines and extracellular matrix in the tumor microenvironment in a way that promotes metastases [239]. A recent tour-de-force study compared the lung microenvironment and behavior of dormant tumor cells in a mouse model of melanoma. This work demonstrated extensive reprogramming of interstitial lung fibroblasts as a consequence of aging, including the upregulation of the WNT pathway antagonist sFRP1; this inhibited the WNT5A produced by dormant tumor cells, leading to DTC awakening to promote metastatic outgrowth [240]. Further investigations of the specific features of the aged lung microenvironment, and how it conditions tumor dormancy, should be of high priority.

As our understanding of the effect of chemotherapy on dormant DTCs and awakening grows, a key impact will be to design studies in which cytotoxic treatments are combined with “anti-awakening therapies.” Designing effective anti-awakening therapies that are sustainable will require careful consideration of tumor signaling pathways, as well as consideration of patient-specific variation in the tumor microenvironment based on age, sex, ancestry, macroenvironmental exposures, and lifestyle. Improved diagnostics would be valuable, to allow the detection of potential micrometastases prior to selection of cancer treatment, and more careful monitoring for metastatic relapse in individuals bearing one or more factors that promote DTC awakening. There is much to be done, but the rapid rate of discovery in this field suggests that control of metastases may indeed be achievable.

## Data Availability

No datasets were generated or analyzed during the current study.
